# Embodied Synaptic Plasticity With Online Reinforcement Learning

**DOI:** 10.3389/fnbot.2019.00081

**Published:** 2019-10-03

**Authors:** Jacques Kaiser, Michael Hoff, Andreas Konle, J. Camilo Vasquez Tieck, David Kappel, Daniel Reichard, Anand Subramoney, Robert Legenstein, Arne Roennau, Wolfgang Maass, Rüdiger Dillmann

**Affiliations:** ^1^FZI Research Center for Information Technology, Karlsruhe, Germany; ^2^Institute for Theoretical Computer Science, Graz University of Technology, Graz, Austria; ^3^Bernstein Center for Computational Neuroscience, III Physikalisches Institut-Biophysik, Georg-August Universität, Göttingen, Germany; ^4^Technische Universität Dresden, Chair of Highly Parallel VLSI Systems and Neuromorphic Circuits, Dresden, Germany

**Keywords:** neurorobotics, synaptic plasticity, spiking neural networks, neuromorphic vision, reinforcement learning

## Abstract

The endeavor to understand the brain involves multiple collaborating research fields. Classically, synaptic plasticity rules derived by theoretical neuroscientists are evaluated in isolation on pattern classification tasks. This contrasts with the biological brain which purpose is to control a body in closed-loop. This paper contributes to bringing the fields of computational neuroscience and robotics closer together by integrating open-source software components from these two fields. The resulting framework allows to evaluate the validity of biologically-plausibe plasticity models in closed-loop robotics environments. We demonstrate this framework to evaluate Synaptic Plasticity with Online REinforcement learning (SPORE), a reward-learning rule based on synaptic sampling, on two visuomotor tasks: reaching and lane following. We show that SPORE is capable of learning to perform policies within the course of simulated hours for both tasks. Provisional parameter explorations indicate that the learning rate and the temperature driving the stochastic processes that govern synaptic learning dynamics need to be regulated for performance improvements to be retained. We conclude by discussing the recent deep reinforcement learning techniques which would be beneficial to increase the functionality of SPORE on visuomotor tasks.

## 1. Introduction

The brain evolved over millions of years for the sole purpose of controlling the body in a goal-directed fashion. Computations are performed relying on neural dynamics and asynchronous communication. Spiking neural network models base their computations on these computational principles. Biologically plausible synaptic plasticity rules for functional learning in spiking neural networks are regularly proposed (Pfister et al., 2006; Urbanczik and Senn, 2014; Neftci, 2017; Kaiser et al., 2018; Zenke and Ganguli, 2018). In general, these rules are derived to minimize a distance (referred to as error) between the output of the network and a target. Therefore, the evaluation of these rules is usually carried out on open-loop pattern classification tasks. By neglecting the embodiment, this type of evaluation disregards the closed-loop dynamics the brain has to handle with the environment. Indeed, the decisions taken by the brain have an impact on the environment, and this change is sensed back by the brain. To get a deeper understanding of the plausibility of these rules, an embodied evaluation is necessary. This evaluation is technically complicated since spiking neurons are dynamical systems that must be synchronized with the environment. Additionally, as in biological bodies, sensory information, and motor commands need to be encoded and decoded respectively.

In this paper, we bring the fields of computational neuroscience and robotics closer together by integrating open-source software components from these two fields. The resulting framework is capable of learning online the control of simulated and real robots with a spiking network in a modular fashion. This framework is demonstrated in the evaluation of the promising neural reward-learning rule SPORE (Kappel et al., 2014, 2015, 2018; Yu et al., 2016) on two closed-loop robotic tasks. SPORE is an instantiation of the synaptic sampling scheme introduced in Kappel et al. (2018, 2015). It incorporates a policy sampling method which models the growth of dendritic spines with respect to dopamine influx. Unlike current state-of-the-art reinforcement learning methods implemented with conventional neural networks (Lillicrap et al., 2015; Mnih et al., 2015, 2016), SPORE learns online from precise spike-time and is entirely implemented with spiking neurons. We evaluate this learning rule in a closed-loop reaching and a lane following (Kaiser et al., 2016; Bing et al., 2018a) setup. In both tasks, an end-to-end visuomotor policy is learned, mapping visual input to motor commands. In the last years, important progress have been made on learning control from visual input with deep learning. However, deep learning approaches are computationally expensive and rely on biologically implausible mechanisms such as dense synchronous communication and batch learning. For networks of spiking neurons learning visuomotor tasks online with synaptic plasticity rules remains challenging. In this paper, visual input is encoded in Address Event Representation with a Dynamic Vision Sensor (DVS) simulation (Lichtsteiner et al., 2008; Kaiser et al., 2016). This representation drastically reduces the redundancy of the visual input as only motion is sensed, allowing more efficient learning. It agrees with the two pathways hypothesis which states that motion is processed separately than color and shape in the visual cortex (Kruger et al., 2013).

The main contribution of this paper is the embodiment of SPORE and its evaluation on two neurorobotic tasks using a combination of open-source software components. This embodiment allowed us to identify crucial techniques to regulate SPORE learning dynamics, not discussed in previous works where this learning rule was only evaluated on simple proof-of-concept learning problems (Kappel et al., 2014, 2015, 2018; Yu et al., 2016). Our results suggest that an external mechanism such as learning rate annealing is beneficial to retain a performing policy on advanced lane following task.

This paper is structured as follows. We provide a review of the related work in section 2. In section 3, we give a brief overview of SPORE and discuss the contributed techniques required for its embodiment. The implementation and evaluation on the two chosen neurorobotic tasks is carried out in section 4. Finally, we discuss in section 5 how the method could be improved.

## 2. Related Work

The year 2015 marked a significant breakthrough in deep reinforcement learning. Artificial neural networks of analog neurons are now capable of solving a variety of tasks ranging from playing video games (Mnih et al., 2015), to controlling multi-joints robots (Lillicrap et al., 2015; Schulman et al., 2017), and lane following (Wolf et al., 2017). Most recent methods (Lillicrap et al., 2015; Schulman et al., 2015, 2017; Mnih et al., 2016) are based on policy-gradients. Specifically, policy parameters are updated by performing ascending gradient steps with backpropagation to maximize the probability of taking rewarding actions. While functional, these methods are not based on biologically plausible processes. First, a large part of neural dynamics are ignored. Importantly, unlike SPORE, these methods do not learn online—weight updates are performed with respect to entire trajectories stored in rollout memory. Second, learning is based on backpropagation which is not biologically plausible learning mechanism, as stated in Bengio et al. (2015).

Spiking network models inspired by deep reinforcement learning techniques were introduced in Bellec et al. (2018) and Tieck et al. (2018). In both papers, the spiking networks are implemented with deep learning frameworks (PyTorch and TensorFlow, respectively) and rely on automatic differentiation. Their policy-gradient approach is based on (PPO; Schulman et al., 2017). As the learning mechanism consists of backpropagating the Proximal Policy Optimization (PPO) loss (through-time in the case of Bellec et al., 2018), most biological constraints stated in Bengio et al. (2015) are still violated. Indeed, the computations are based on spikes (4), but the backpropagation is purely linear (1), the feedback paths require precise knowledge of the derivatives (2) and weights (3) of the corresponding feedforward paths, and the feedforward and feedback phases alternate synchronously (5) (the enumeration refers to Bengio et al., 2015).

Only a small body of work focused on reinforcement learning with spiking neural networks, while addressing the previous points. Groundwork of reinforcement learning with spiking networks was presented in Florian (2007), Izhikevich (2007), and Legenstein et al. (2008). In these works, a mathematical formalization is introduced characterizing how dopamine modulated spike-timing-dependent plasticity (DA-STDP) solves the distal reward problem with eligibility traces. Specifically, since the reward is received only after a rewarding action is performed, the brain needs a form of memory to reinforce previously chosen actions. This problem is solved with the introduction eligibility traces, which assign credit to recently active synapses. This concept has been observed in the brain (Frey and Morris, 1997; Pan et al., 2005), and SPORE also relies on eligibility traces. Fewer works evaluated DA-STDP in an embodiment for reward maximization—a recent survey encompassing this topic is available in Bing et al. (2018b).

The closest previous work related to this paper are Daucé (2009), Kaiser et al. (2016), and Bing et al. (2018a). In Kaiser et al. (2016), a neurorobotic lane following task is presented, where a simulated vehicle is controlled end-to-end from event-based vision to motor command. The task is solved with an hard-coded spiking network of 16 neurons implementing a simple Braitenberg vehicle. The performance is evaluated with respect to distance and orientation differences to the middle of the lane. In this paper, these performance metrics are combined into a reward signal which the spiking network maximizes with the SPORE learning rule.

In Bing et al. (2018a), the authors evaluate DA-STDP (referred to as R-STDP for reward-modulated STDP) in a similar lane following environment. Their approach outperforms the hard-coded Braitenberg vehicle presented in Kaiser et al. (2016). The two motor neurons controlling the steering receive different (mirrored) reward signals whether the vehicle is on the left or on the right of the lane. This way, the reward provides the information of what motor command should be taken, similar to a supervised learning setup. Conversely, the approach presented in this paper is more generic since a global reward is distributed to all synapses and does not indicate which action the agent should take.

A similar plasticity rule implementing a policy-gradient approach is derived in Daucé (2009). Also relying on eligibility traces, this reward-learning rule uses a “slow” noise term to drive the exploration. This rule is demonstrated on a target reaching task comparable to the one discussed in section 4.1.1 and achieves impressive learning times (in the order of 100s) with proper tuning of the noise term.

In Nakano et al. (2015), a spiking version of the free-energy-based reinforcement learning framework proposed in Otsuka et al. (2010) is introduced. In this framework, a spiking Restricted Boltzmann Machine (RBM) is trained with a reward-modulated plasticity rule which decreases the free-energy of rewarding state-action pairs. The approach is evaluated on discrete-actions tasks where the observations consist of MNIST digits processed by a pre-trained feature extractor. However, some characteristics of RBM are biologically implausible and make their implementation cumbersome: symmetric synapses and clocked network activity. With our approach, network activity does not have to be manually synchronized into observation and action phases of arbitrary duration for learning to take place.

In Gilra and Gerstner (2017), a supervised synaptic learning rule named Feedback-based Online Local Learning Of Weights (FOLLOW) is introduced. This rule is used to learn the inverse dynamics of a two-link arm—the model predicts control commands (torques) for a given arm trajectory. The loop is closed in Gilra and Gerstner (2018) by feeding the predicted torques as control commands. In contrast, SPORE learns from a reward signal and can solve a variety of tasks.

## 3. Methods

In this section, we give a brief overview of the reward-based learning rule SPORE. We then discuss how SPORE was embodied in closed-loop, along with our modifications to increase the robustness of the learned policy.

### 3.1. Synaptic Plasticity With Online Reinforcement Learning (SPORE)

Throughout our experiments we use an implementation of the reward-based online learning rule for spiking neural networks, named *synaptic sampling*, that was introduced in Kappel et al. (2018). The learning rule employs synaptic updates that are modulated by a global reward signal to maximize the expected reward. More precisely, the learning rule does not converge to a local maximum θ^*^ of the synaptic parameter vector ***θ***, but it continuously samples different solutions ***θ*** ~ p^*^(***θ***) from a target distribution that peaks at parameter vectors that likely yield high reward. A temperature parameter *T* allows to make the distribution p^*^(***θ***) flatter (high exploration) or more peaked (high exploitation).

SPORE (Kappel et al., 2017) is an implementation of the reward-based synaptic sampling rule (Kappel et al., 2018), that uses the NEST neural simulator (Gewaltig and Diesmann, 2007). SPORE is optimized for closed-loop applications to form an online policy-gradient approach. We briefly review here the main features of the synaptic sampling algorithm.

We consider the goal of reinforcement learning to maximize the expected future discounted reward V(θ) given by

(1) $$\begin{array}{l} V\left(\theta \right)={\langle \int_{0}^{\infty }e^{-\frac{\tau }{\tau_{e}}}\;r\left(\tau \right)\;d\tau \rangle }_{p\left(\text{r}|\theta \right)}, \end{array}$$

where *r*(τ) denotes the reward at time τ and τ_*e*_ is a time constant that discounts remote rewards. We consider non-negative reward *r*(τ) ≥ 0 at any time such that V(θ)≥0 for all ***θ***. The distribution *p*(***r***|***θ***) denotes the probability of observing the sequence of reward ***r*** under a given parameter vector ***θ***. Note that computing this expectation involves averaging over a number of experimental trials and network responses.

As proposed in Kappel et al. (2018) we replace the standard goal of reinforcement learning to maximize the objective function in Equation (1) by a probabilistic framework that generates samples from the parameter vector ***θ*** according to some target distribution ***θ*** ~ p^*^(***θ***). We will focus on sampling from the target distribution p^*^(***θ***) of the form

(2) $$\begin{array}{l} p^{*}\left(\theta \right)\propto p\left(\theta \right)\times V\left(\theta \right), \end{array}$$

where *p*(***θ***) is a prior distribution over the network parameters that allows us, for example, to introduce constraints on the sparsity of the network parameters. It has been shown in Kappel et al. (2018) that the learning goal in is achieved, if all synaptic parameters *θ*_*i*_ obey the stochastic differential equation

(3) $$\begin{array}{l} d\theta_{i}=\beta \left(\frac{\partial }{\partial \theta_{i}}\log p\left(\theta \right)+\frac{\partial }{\partial \theta_{i}}\log V\left(\theta \right)\right)\;dt+\sqrt{2\beta T}\;dW_{i}, \end{array}$$

where *β* is a scaling parameter that functions as a learning rate, $dW_{i}$ are the stochastic increments and decrements of a Wiener process, and *T* is the temperature parameter. $\frac{\partial }{\partial \theta_{i}}$ denotes the partial derivative with respect to the synaptic parameter *θ*_*i*_. The stochastic process in generates samples of ***θ*** that are with high probability close to the local optima of the target distribution p^*^(***θ***).

It has been further shown in Kappel et al. (2018) that can be implemented using a synapse model with local update rules. The state of each synapse *i* consists of the dynamic variables *y*_*i*_(*t*), *e*_*i*_(*t*), *g*_*i*_(*t*), θ_*i*_(*t*), and *w*_*i*_(*t*). The variable *y*_*i*_(*t*) is the pre-synaptic spike train filtered with a post-synaptic-potential kernel. *e*_*i*_(*t*) is the eligibility trace that maintains a brief history of pre-/post neural activity. *g*_*i*_(*t*) is a variable to estimate the reward gradient, i.e., the gradient of the objective function in Equation (1) with respect to the synaptic parameter θ_*i*_(*t*). *w*_*i*_(*t*) denotes the weight of synapse *i* at time *t*. In addition each synapse has access to the global reward signal *r*(*t*). The variables *e*_*i*_(*t*), *g*_*i*_(*t*), and θ_*i*_(*t*) are updated by solving the differential equations:

(4) $$\begin{array}{l} \frac{de_{i}\left(t\right)}{dt}=-\frac{1}{\tau_{e}}e_{i}\left(t\right)+w_{i}\left(t\right)y_{i}\left(t\right)\left(z_{post_{i}}\left(t\right)-\rho_{post_{i}}\left(t\right)\right) \end{array}$$

(5) $$\begin{array}{l} \frac{dg_{i}\left(t\right)}{dt}=-\frac{1}{\tau_{g}}g_{i}\left(t\right)+r\left(t\right)e_{i}\left(t\right) \end{array}$$

(6) $$\begin{array}{l} d\theta_{i}\left(t\right)=\beta (c_{p}\left(\mu -\theta_{i}\left(t\right)\right)+c_{g}g_{i}\left(t\right))dt+\sqrt{2T_{\theta }\beta }W_{i}, \end{array}$$

where *z*_*post*_*i*__(*t*) is a sum of Dirac delta pulses placed at the firing times of the post-synaptic neuron, μ is the prior mean of synaptic parameters [*p*(***θ***) in Equation 2] and ρ_*post*_*i*__(*t*) is the instantaneous firing rate of the post-synaptic neuron at time *t*. The constants *c*_*p*_ and *c*_*g*_ are tuning parameters of the algorithm that scale the influence of the prior distribution *p*(***θ***) against the influence of the reward-modulated term. Setting *c*_*p*_ = 0 corresponds to a non-informative (flat) prior. In general, the prior distribution is modeled as a Gaussian centered around μ: $p\left(\theta \right)=N\left(\mu ,\frac{1}{c_{p}}\right)$. We used μ = 0 in our simulations. The variance of the reward gradient estimation (Equation 5) could be reduced by subtracting a baseline to the reward as introduced in Williams (1992), although this was not investigated in this paper.

Finally the synaptic weights are given by the projection

(7) $$\begin{array}{l} w_{i}(t)\;=\;\{\begin{array}{ll} w_{0}\exp(\theta_{i}(t)-\theta_{0}) & \text{ if }\theta_{i}(t)>0\\ 0 & \text{ otherwise} \end{array}\;, \end{array}$$

which scaling and offset parameters *w*_0_ and θ_0_, respectively.

In SPORE the differential equations Equations (4) to (6) are solved using the Euler method with a time step of 1 ms. The dynamics of the post-synaptic term *y*_*i*_(*t*), the eligibility trace *e*_*i*_(*t*), and the reward gradient *g*_*i*_(*t*) are updated at each time step. The dynamics of θ_*i*_(*t*) and *w*_*i*_(*t*) are updated on a coarser time grid with step width 100 ms for the sake of simulation speed. The synaptic weights remain constant between two updates. Synaptic parameters are clipped at θ_*min*_ and θ_*max*_. Parameter gradients *g*_*i*_(*t*) are clipped at ±Δθ_*max*_. The parameters used in our evaluation are stated in Tables 1–3.

**Table 1:** NEST parameters.

Time-step/resolution	1 ms
Synapse update interval	100 ms
(reaching) exploration noise	35 Hz
(reaching) noise to exploration exc.	750.0
(reaching) visual to exploration inh.	N(-500,50)
(reaching) exploration to motor exc.	10.0

**Table 2:** SPORE parameters.

Visual to motor exc.	N(0.8,0.6) (clipped at 0)
Visual to motor mul.	10
Temperature (*T*)	0.1
Initial learning rate (β)	1 × 10^−7^
Learning rate decay (λ)	8.5 × 10^−5^
Integration time	50 s
Max synaptic parameter (θ_*max*_)	5.0
Min synaptic parameter (θ_*min*_)	−2.0
(reaching) episode length	1 s
(lane following) episode length	2 s

**Table 3:** ROS-MUSIC parameters.

MUSIC time-step	1 ms…3 ms
DVS adapter time-step	1 ms
Decoder time constant	100 ms

### 3.2. Closed-Loop Embodiment Implementation

**Figure 1:**
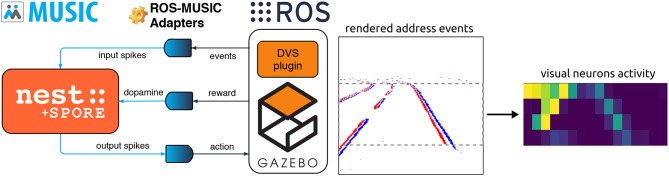
Implementation of the embodied closed-loop evaluation of the reward-based learning rule SPORE. **(Left)** Our asynchronous framework based on open-source software components. The spiking network is implemented with the NEST neural simulator (Gewaltig and Diesmann, 2007), which communicates spikes with MUSIC (Ekeberg and Djurfeldt, 2008; Djurfeldt et al., 2010). The reward is streamed to all synapses in the spiking network learning with SPORE (Kappel et al., 2017). Spikes are encoded from address events and decoded to motor commands with ROS-MUSIC tool-chain adapters (Weidel et al., 2016). Address events are emitted by the DVS plugin (Kaiser et al., 2016) within the simulated robotic environment Gazebo (Koenig and Howard, 2004), which communicates with ROS (Quigley et al., 2009). **(Right)** Encoding visual information to spikes for the lane following experiment, see section 4.1.2 for more information. Address events (red and blue pixels on the rendered image) are downscaled and fed to visual neurons as spikes.

Usually, synaptic learning rules are solely evaluated on open-loop pattern classification tasks (Pfister et al., 2006; Urbanczik and Senn, 2014; Neftci, 2017; Zenke and Ganguli, 2018). An embodied evaluation is technically more involved and requires a closed-loop environment simulation. A core contribution of this paper is the implementation of a framework allowing to evaluate the validity of bio-plausibe plasticity models in closed-loop robotics environments. We rely on this framework to evaluate the synaptic sampling rule SPORE (Kappel et al., 2017), as depicted in Figure 1. This framework is tailored for evaluating spiking network learning rules in an embodiment. Visual sensory input is sensed, encoded as spikes, processed by the network, and output spikes are converted to motor commands. The motor commands are executed by the agent, which modifies the environment. This modification of the environment is sensed by the agent. Additionally, a continuous reward signal is emitted from the environment. SPORE tries to maximize this reward signal online by steering the ongoing synaptic plasticity processes of the network toward configurations which are expected to yield more overall reward. Unlike classical reinforcement learning setup, the spiking network is treated as a dynamical system continuously receiving input and outputting motor commands. This allows us to report learning progress with respect to (biological) simulated time, unlike classical reinforcement learning which reports learning progress in number of iterations. Similarly, we reset the agent only when the task is completed (in the reaching task) or when the agent goes off-track (in the lane following task). We do not enforce finite-time episodes and neither the agent nor SPORE are notified of the reset.

This framework relies on many open-source software components: As neural simulator we use NEST (Gewaltig and Diesmann, 2007) combined with the open-source implementation of SPORE (Kappel et al., 2018)^1^. The robotic simulation is managed by Gazebo (Koenig and Howard, 2004) and ROS (Quigley et al., 2009) and visual perception is realized using the open-source DVS plugin for Gazebo (Kaiser et al., 2016)^2^. This plugin emits polarized address events when variations in pixel intensity cross a threshold. The robotic simulator and the neural network run in different processes. We rely on MUSIC (Ekeberg and Djurfeldt, 2008; Djurfeldt et al., 2010) to communicate and transform the spikes and we employ the ROS-MUSIC tool-chain by Weidel et al. (2016) to bridge between the two communication frameworks. The latter also synchronizes ROS time with spiking network time. Most of these components are also integrated in the Neurorobotics Platform (NRP) Falotico et al. (2017), except for MUSIC and the ROS-MUSIC tool-chain. Therefore, the NRP does not support streaming a reward signal to all synapses, required in our experiments.

As part of this work, we contributed to the Gazebo DVS plugin by integrating it to ROS-MUSIC, and to the SPORE module by integrating it with MUSIC. These contributions enable researchers to design new ROS-MUSIC experiments using event-based vision to evaluate SPORE or their own biologically-plausible learning rules. A clear advantage of this framework is that the robotic simulation can be substituted for a real robot seamlessly. However, the necessary human supervision in real robotics coupled with the many hours needed by SPORE to learn a performing policy is currently prohibitive. The simulation of the whole framework was conducted on a Quad core Intel Core i7-4790K with 16GB RAM in real-time.

### 3.3. Learning Rate Annealing

In the original work presenting SPORE (Kappel et al., 2014, 2015, 2018; Yu et al., 2016), the learning rate β and the temperature *T* were kept constant throughout the learning process. Note that in deep learning, learning rates are often regulated by the optimization processes (Kingma and Ba, 2014). We found that the learning rate β of SPORE plays an important role in learning and benefit from an annealing mechanism. This regulation allows the synaptic weights to converge to a stable configuration and prevents the network to forget previous policy improvements. For the lane following experiment presented in this paper, the learning rate β is decreased over time, which also reduces the temperature (random exploration), see Equation (3). Specifically, we decay the learning rate β exponentially with respect to time:

(8) $$\begin{array}{l} \frac{d\beta \left(t\right)}{dt}=-\lambda \beta \left(t\right). \end{array}$$

The learning rate is updated following this equation every 10 min. Independently decaying the temperature term *T* was not investigated, however we expect a minor impact on the performance because of the high variance of the reward gradient estimation, intrinsically leading the agent to explore.

## 4. Evaluation

We evaluate our approach on two neurorobotic tasks: a reaching task and the lane following task presented in Kaiser et al. (2016) and Bing et al. (2018a). In the following sections, we describe these tasks and the ability of SPORE to solve them. Additionally, we analyze the performance and stability of the learned policies with respect to the prior distribution *p*(***θ***) and learning rate β (see Equation 3).

### 4.1. Experimental Setup

**Figure 2:**
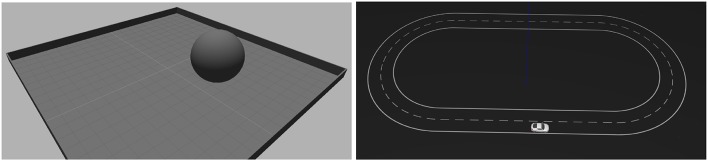
Visualization of the setup for the two experiments. **(Left)** Reaching experiment. The goal of the task is to control the ball to the center of the plane. Visual input is provided by a DVS simulation above the plane looking downward. The ball is controlled with Cartesian velocity vectors. **(Right)** Lane following experiment. The goal of the task is to keep the vehicle on the right lane of the road. Visual input is provided by a DVS simulation attached to the vehicle looking forward to the road. The vehicle is controlled with steering angles.

The tasks used for our evaluation are depicted in Figure 2. In both tasks, a feed-forward all-to-all two-layers network of spiking neurons is trained with SPORE to maximize a task-specific reward. Previous work has shown that this architecture was sufficient for the task complexity considered (Daucé, 2009; Kaiser et al., 2016; Bing et al., 2018a). The network is end-to-end and maps the address events of a simulated DVS to motor commands. The parameters used for the evaluation are presented in Tables 1–3. In the next paragraphs, we describe the tasks together with their decoding schemes and reward functions.

#### 4.1.1. Reaching Task

The reaching task is a natural extension of the open-loop blind reaching task on which SPORE was evaluated in Yu et al. (2016). A similar visual tracking task was presented in Daucé (2009), with a different visual input encoding. In our setup, the agent controls a ball of 2 m radius which has to move toward the 2 m radius center of a 20 × 20 m plane enclosed with walls. Sensory input is provided by a simulated DVS with a resolution of 16x16 pixels located above the center which perceives the ball and the entire plane. There is one visual neuron corresponding to each DVS pixel—we make no distinctions between ON and OFF events. We additionally enhance the input space with an axis feature neuron for each row and each column. These neurons fire for each spikes in the respective row or column of neurons they cover. Both 16x16 visual neurons and 2x16 axis feature neurons are connected to all 8 motor neurons with 10 plastic SPORE synapses, resulting in 23,040 learnable parameters. The network controls the ball with instantaneous velocity vectors through the Gazebo Planar Move Plugin. Velocity vectors are decoded from output spikes with the linear decoder:

(9) $$\begin{array}{l} \begin{array}{c} v=\left[\begin{array}{c} ẋ\\ ẏ \end{array}\right]=\left[\begin{array}{cccc} cos\left(\beta_{1}\right) & cos\left(\beta_{2}\right) & \ldots & cos\left(\beta_{N}\right)\\ sin\left(\beta_{1}\right) & sin\left(\beta_{2}\right) & \ldots & sin\left(\beta_{N}\right) \end{array}\right]\left[\begin{array}{c} a_{1}\\ a_{2}\\ \vdots \\ a_{N} \end{array}\right]\\ \beta_{k}=\frac{2k\pi }{N}, \end{array} \end{array}$$

with *a*_*k*_ the activity of motor neuron *k* obtained by applying a low-pass filter on the spikes with time constant τ. This decoding scheme consists of equally distributing *N* motor neurons on a circle representing their contribution to the displacement vector. For our experiment, we set *N* = 8 motor neurons. We add an additional exploration neuron to the network which excites the motor neurons and is inhibited by the visual neurons. This neuron prevents long periods of immobility. Indeed, when the agent decides to stay motionless, it does not receive any sensory input as the DVS simulation only senses change. Since the network is feedforward, the absence of sensory input causes the neural activity to drop, leading to more immobility.

The ball is reset to a random position on the plane if it has reached the center. This reset is not signaled to the network—aside from the abrupt change in visual input—and does not mark the end of an episode. Let β_err_ denote the absolute value of the angle between the straight line to the goal and the direction taken by the ball. The agent is rewarded if the ball moves in the direction toward the goal β_err_ < β_lim_ at a sufficient velocity *v* > *v*_lim_. Specifically, the reward *r*(*t*) is computed as:

(10) $$\begin{array}{l} r(t)=35\sqrt{r_{v}}{\left(r_{\beta }+1\right)}^{5}\\ \;r_{\beta }=\{\begin{array}{cc} 1-\frac{\beta_{\text{err}}}{\beta_{\text{lim}}}, & \text{if }\beta_{\text{err}}<\beta_{\text{lim}}\\ 0, & \text{otherwise} \end{array}\\ \;r_{v}=\{\begin{array}{ll} |v|, & \text{if }|v|>v_{\text{lim}}\\ 0, & \text{otherwise} \end{array}. \end{array}$$

This signal is smoothed with an exponential filter before being streamed to the agent. This formulation provides a continuous feedback to the agent, unlike delivering a discrete terminal reward upon reaching the goal state. In our experiments, discrete terminal rewards did not suffice for the agent to learn performing policies in a reasonable amount of time. On the other hand, distal rewards are supported by SPORE through eligibility traces, as was demonstrated in Yu et al. (2016) and Kappel et al. (2018), for open-loop tasks with clearly delimited episodes. This suggests that additional mechanisms or hyperparameter tuning would be required for SPORE to learn from distal rewards online.

#### 4.1.2. Lane Following Task

The lane following task was already used to demonstrate spiking neural controllers in Kaiser et al. (2016) and Bing et al. (2018a). The goal of the task is to steer a vehicle to stay on the right lane of a track. Sensory input is provided by a simulated DVS with a resolution of 128x32 pixels mounted on top of the vehicle showing the track in front. There are 16x4 visual neurons covering the pixels, each neuron responsible for a 8x8 pixel window. Each visual neuron spikes at a rate correlated to the amount of events in its window (see Figure 1). The vehicle starts driving on a fixed starting point with a constant velocity on the right lane of the track. As soon as the vehicle leaves the track, it is reset to the starting point. As in the reaching task, this reset is not explicitly signaled to the network and does not mark the end of a learning episode.

The network controls the angle of the vehicle by steering it, while its linear velocity is constant. The output layer is separated into two neural populations. The steering commands sent to the agent consist of the difference of activity between these two populations. Specifically, steering commands are decoded from output spikes as a ratio between the following linear decoders:

(11) $$\begin{array}{l} a_{L}=\overset{N/2}{\underset{i=1}{\sum }}a_{i},\\ a_{R}=\overset{N}{\underset{i=N/2}{\sum }}a_{i},\\ r=\frac{a_{L}-a_{R}}{a_{L}+a_{R}}. \end{array}$$

The first *N*/2 neurons pull the steering on one side, while the remaining *N*/2 neurons pull steering to the other side. We set *N* = 8 so that there are 4 left motor neurons and 4 right motor neurons. The steering command is obtained by discretizing the ratio *r* into five possible commands: hard left (–30°), left (–15°), straight (0°), right (15°), and hard right (30°). The decision boundaries between these steering angles are *r* = {−10, −2.5, 2.5, 10}, respectively. This discretization is similar than the one used in Wolf et al. (2017). It yielded better performance than directly using *r* (multiplied with a scaling constant *k*) as a continuous-space steering command as in Kaiser et al. (2016).

The reward signal delivered to the vehicle is equivalent to the performance metrics used in Kaiser et al. (2016) to evaluate the policy. As in the reaching task, the reward depends on two terms—the angular error β_err_ and the distance error *d*_err_. The angular error β_err_ is the absolute value of the angle between the right lane and the vehicle. The distance error *d*_err_ is the distance between the vehicle and the center of the right lane. The reward *r*(*t*) is computed as:

(12) $$\begin{array}{l} r\left(t\right)=e^{-0.03\;\beta_{\text{err}}^{2}}\times e^{-70\;d_{\text{err}}^{2}}. \end{array}$$

The constants are chosen so that the score is halved every 0.1m distance error or 5° angular error. Note that this reward function is comprised between [0, 1] and is less informative than the error used in Bing et al. (2018a). In our case, the same reward is delivered to all synapses, and a particular reward value does not indicate whether the vehicle is on the left or on the right of the lane. The decay of the learning rate is λ = 8.5 × 10^−5^ (see Table 2).

### 4.2. Results

Our results show that SPORE is capable of learning policies online for moderately difficult embodied tasks within some simulated hours (see Supplementary Video). We first discuss the results on the reaching task, where we evaluated the impact of the prior distribution. We then present the results on the lane following task, where the impact of the learning rate was evaluated.

#### 4.2.1. Impact of Prior Distribution

**Figure 3:**
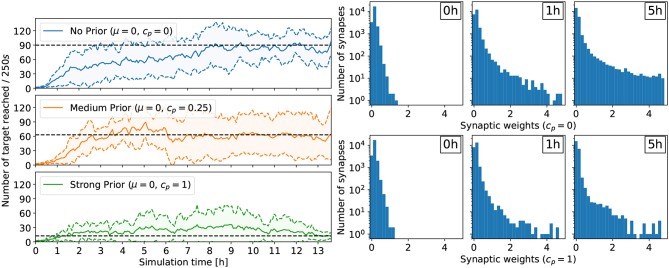
Results for the reaching task. **(Left)** Comparing the effect of different prior configurations on the overall learning performance. The results were averaged over eight trials. The performance is measured with the rate at which the target is reached (the ball moves to the center and is reset at a random position). **(Right)** Development of the synaptic weights over the course of learning for two trials: no prior (*c*_*p*_ = 0, top) and strong prior (*c*_*p*_ = 1, bottom). In both cases, the number of weak synaptic weights (below 0.07) increases significantly over time.

For the reaching task, a flat prior *c*_*p*_ = 0 yielded the policy with highest performance (see Figure 3). In this case, the performance improves rapidly within a few hours of simulated time, and the ball reaches the center about 90 times every 250 s. Conversely, a strong prior (*c*_*p*_ = 1) forcing the synaptic weights close to 0 prevented performing policies to emerge. In this case, after 13h of learning, the ball reaches the center only about 10 times on average every 250 s, a performance comparable to the random policy. Less constraining priors also affected the performance of the learned policies compared to the unconstrained case, but allowed learning to happen. With *c*_*p*_ = 0.25, the ball reaches the center about 60 times on average every 250 s. Additionally, the number of retracting synapses increases over time—even in the flat prior case—reducing the computational overhead, important for a neuromorphic hardware implementation (Bellec et al., 2017). Indeed, for *c*_*p*_ = 0, the number of weak synaptic weights (below 0.07) increased from 3,329 to 7,557 after 1h of learning to 14,753 after 5 h of learning (out of 23,040 synapses in total). In other words, only 36% of all synapses are active. The weight distribution for *c*_*p*_ = 0.25 is similar to the no-prior case *c*_*p*_ = 0. The strong prior *c*_*p*_ = 1 prevented strong weights to form, trading-off performance. The same trend is observed for the lane following task, where only 33% of all synapses are active after 4 h of learning (see **Figure 5**).

**Figure 4:**
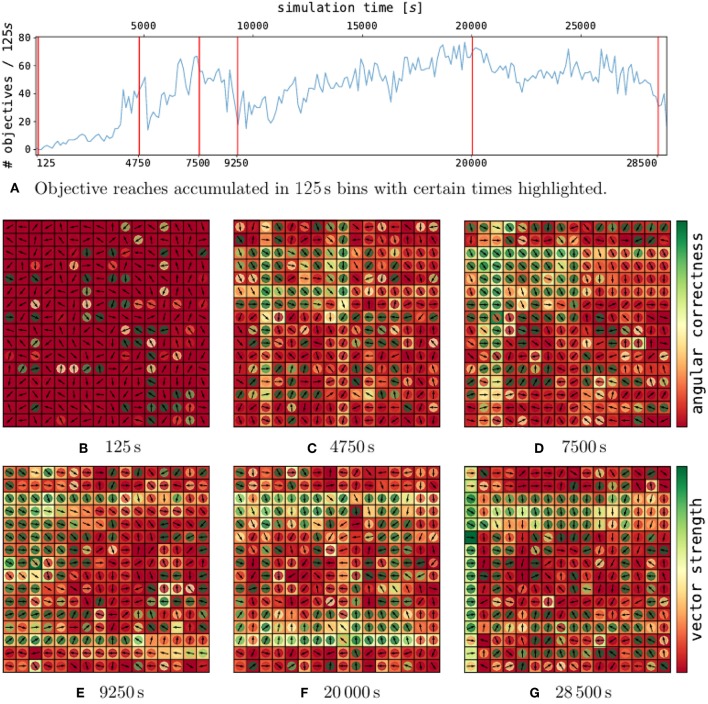
Policy development for selected points in time in a single trial. On the top **(A)**, the performance over time for a single, well-performing trial is depicted. The red lines indicate certain points in time, for which the policies are shown in **(B–G)**. Each policy plot consists of a 2d-grid representing the DVS pixels. Hereby, every pixel contains a vector, which indicates the motion corresponding to the contribution of an event emitted by this pixel. The magnitude of the contribution (vector strength) is indicated by the outer pixel area. The inner circle color represents the assessment of the vector direction (angular correctness).

The analysis of a single trial with *c*_*p*_ = 0.25 is depicted in Figure 4. The performance does not converge and rather rise and drop while the network is sampling configurations. On initialization (Figure 4B), the policy employs weak actions with random directions.

After over 4.750 s of learning (Figure 4C), the first local maximum is reached. Vector directions have largely turned toward the grid center (see inner pixel colors). Additionally, the overall magnitude of the weights has largely increased, as could be expected from the weight histogram in Figure 3. In particular, patterns of single rows and columns emerge, due to the 2x16 axis feature neurons described in section 4.1.1. One drawback of the axis feature neurons can be seen in the center column of pixel. The axis feature neuron responsible for this column learned to push the ball down, since the ball mostly visited the upper part of the grid. However, at the center, the correct direction to push the ball toward the center is flipped.

At 7.500 s (Figure 4D), the performance has further increased. The policy, as shown in the second peak has grown even stronger for many pixels which also point in the right direction. The pixels pointing in the wrong direction mostly have a low vector strength.

After 9.250 s (Figure 4E), the performance drops to half its previous performance. As we can see from the policy, the weights grew even stronger. Some strong pixels vectors pointing toward each other have emerged, which can lead to the ball constantly moving up and down, without receiving any reward.

After this valley, the performance rises slowly again and at 20 000 s of simulation time (Figure 4F) the policy has reached the maximum performance of this trial. Around the whole grid, strong motion vectors push the ball toward the center, and the ball reaches the center around 140 times every 250 s.

Just before the end of the trial, the performance drops again (Figure 4G). Most vectors still point toward the right direction, however, the overall strength has largely decreased.

#### 4.2.2. Impact of Learning Rate

**Figure 5:**
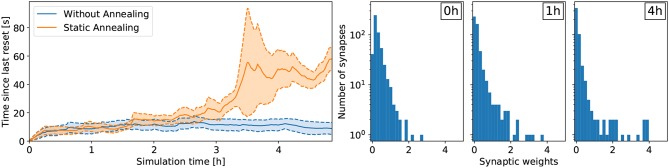
Results for the lane following task with a medium prior (*c*_*p*_ = 0.25). **(Left)** Comparing the effect of annealing on the overall learning performance. The results were averaged over six trials. Without annealing, performance improvements are not retained and the network does not learn to perform the task. With annealing, the learning rate β decreases over time and performance improvements are retained. **(Right)** Development of the synaptic weights over the course of learning for a medium prior of *c*_*p*_ = 0.25 with annealing. The number of weak synaptic weights (below 0.07) increases from 41 to 231 after 1h of learning to 342 after 4 h of learning (out of 512 synapses in total).

For the lane following experiment, we show that the learning rate β plays an important role for retaining policy improvements. Specifically, when the learning rate β remains constant over the course of learning, the policy does not improve compared to random (see Figure 5). In the random case, the vehicle remains about 10 s on the right lane until triggering a reset. After about 3 h of learning, the learning rate β decreased to 40% of its initial value and the policy starts to improve. After 5 h of learning, the learning rate β approaches 20% of its initial value and the performance improvements are retained. Indeed, while the weights are not frozen, the amplitude of subsequent synaptic updates are drastically reduced. In this case, the policy is significantly better than random and the vehicle remains on the right lane about 60 s on average.

## 5. Conclusion

The endeavor to understand the brain spans over multiple research fields. Collaborations allowing synaptic learning rules derived by theoretical neuroscientists to be evaluated in closed-loop embodiment are an important milestone of this endeavor. In this paper, we successfully implemented a framework allowing this evaluation by relying on open-source software components for spiking network simulation (Gewaltig and Diesmann, 2007; Kappel et al., 2017), synchronization and communication (Ekeberg and Djurfeldt, 2008; Quigley et al., 2009; Djurfeldt et al., 2010; Weidel et al., 2016), and robotic simulation (Koenig and Howard, 2004; Kaiser et al., 2016). The resulting framework is capable of learning online the control of simulated and real robots with a spiking network in a modular fashion. This framework is used to evaluate the reward-learning rule SPORE (Kappel et al., 2014, 2015, 2018; Yu et al., 2016) on two closed-loop visuomotor tasks. Overall, we have shown that SPORE was capable of learning shallow feedforward policies online for moderately difficult embodied tasks within some simulated hours. This evaluation allowed us to characterize the influence of the prior distribution on the learned policy. Specifically, constraining priors deteriorate the performance of the learned policy but prevent strong synaptic weights to emerge (see Figure 3). Additionally, for the lane following experiment, we have shown how learning rate regulation enabled policy improvements to be retained. Inspired by simulated annealing, we presented a simple method decreasing the learning rate over time. This method does not model a particular biological mechanism, but seems to work better in practice. On the other hand, novelty is known to modulate plasticity through a number of mechanisms (Hamid et al., 2016; Rangel-Gomez and Meeter, 2016). Therefore, a decrease in learning rate after familiarization with the task is reasonable.

On a functional scale, deep learning methods still outperform biologically plausible learning rules such as SPORE. For future work, the performance gap between SPORE and deep learning methods should be tackled by taking inspiration from deep learning methods. Specifically, the online learning method inherent to SPORE is impacted by the high variance of the policy evaluation. This problem was alleviated in policy-gradient methods by introducing a critic trained to estimate the expected return of a given state. This expected return is used as a baseline which reduces the variance of the policy evaluation. Decreasing the variance could also be achieved by considering an action-space noise as in Daucé (2009) instead of a parameter-space noise implemented by the Wiener process in . Lastly, an automatic mechanism to regulate the learning rate β is beneficial for more complex task. Such a mechanism could be inspired by trust-region methods (Schulman et al., 2015), which constrains weight updates to alter the policy little by little. These improvements should increase SPORE performance so that more complex tasks such as multi-joint effector control and discrete terminal rewards—supported by design by the proposed framework—could be considered.

## Author Contributions

All the authors participated in writing the paper. JK, MH, AK, JV, and DK conceived the experiments and analyzed the data.

### Conflict of Interest

The authors declare that the research was conducted in the absence of any commercial or financial relationships that could be construed as a potential conflict of interest.

## Footnotes

^1^https://github.com/IGITUGraz/spore-nest-module

^2^https://github.com/HBPNeurorobotics/gazebo_dvs_plugin
